# Techniques and future perspectives for the prevention and treatment of endoleaks after endovascular repair of abdominal aortic aneurysms

**DOI:** 10.1186/s13244-019-0774-y

**Published:** 2019-09-23

**Authors:** Gianluigi Orgera, Marcello Andrea Tipaldi, Florindo Laurino, Pierleone Lucatelli, Alberto Rebonato, Ioannis Paraskevopoulos, Michele Rossi, Miltiadis Krokidis

**Affiliations:** 1 Department of Radiology, Sant’ Andrea University Hospital La Sapienza, Rome, Italy; 2 Department of Radiological Sciences, Sapienza University of Rome, Rome, Italy; 3 The Department of Surgical and Biomedical Sciences, University of Perugia, Perugia, Italy; 4 The Department of Radiology, Aberdeen Royal Infirmary, NHS Grampian, Aberdeen, UK; 5 Department of Radiology, Cambridge University Hospitals NHS Foundation Trust, Hills Road, Cambridge, CB2 0QQ UK

**Keywords:** Aneurysm, Endoleak, Aorta

## Abstract

The presence of endoleaks remains one of the main drawbacks of endovascular repair of abdominal aortic aneurysms leading to the increase of the size of the aneurysmal sac and in most of the cases to repeated interventions. A variety of devices and percutaneous techniques have been developed so far to prevent and treat this phenomenon, including sealing of the aneurysmal sac, endovascular embolisation, and direct sac puncture. The aim of this review is to analyse the indications, the effectiveness, and the future perspectives for the prevention and treatment of endoleaks after endovascular repair of abdominal aortic aneurysms.

## Key points

- The detection rate of endoleaks depends on the imaging modalities used
- Only a small percentage of endoleaks will require re-intervention
- Treatment may include both endovascular or percutaneous route

## Introduction

The endovascular aortic repair (EVAR) of abdominal aortic aneurysms was first described nearly three decades ago and has offered a crucial management shift of patients with aortic disease, particularly when open repair was not an option [1–3].

A variety of EVAR devices were developed over the years offering a range of outcomes. There has been a substantial evolution in design and technology, from the initial tube grafts to the custom-made fenestrated and branched devices that are used today. EVAR has offered some benefits over the traditional open surgical repair; however, there is a cost to pay and this is mainly the need of closer patient follow-up and sometimes the necessity of re-interventions [4–9]. Follow-up is required to assess growth of the aneurysm sac, device migration, blockage, or infection. The most common reason for re-intervention is the increase of the aneurysmal sac due persistence of flow, a phenomenon otherwise known as “endoleak” [4–9]. The purpose of this review article is to illustrate the various types of endoleaks and to describe what the current status of percutaneous management is.

## Classification of endoleaks

Endoleaks are classified into five types (I–V). *Type I* occurs due to incomplete proximal (Ia) or distal (Ib) seal. This could be due to either inappropriate device selection, incorrect graft deployment, or disease progression [10]. Type Ia may also appear when chimneys are used and are known as “gutter endoleaks” [11, 12]. *Type II* occurs due to sac centripetal reperfusion via side branches (lumbar arteries, inferior mesenteric artery, accessory renal arteries) with inverted flow. *Type III* is a result of dislodgement of the various graft components. *Type IV* occurs due to increased porosity of the graft material. *Type V* (also known as “endotension”) is the type of endoleak that cannot be classified in any of the other categories.

## Incidence

It is not easy to define the precise incidence of endoleaks from the existing data in the literature. The evidence-based practice centre of the Mayo Clinic has published in 2017 a systematic review and meta-regression analysis evaluating surveillance outcomes after EVAR for AAA including 6 meta-analyses and 52 observational studies [13]. The authors confirmed that an endoleak incidence rate is subject to the type of imaging modality used for their detection and reported that a combined approach of DUS, non-contrast-enhanced CT, and MRI offered the highest endoleak detection rate at 12, 24, 36, and 48 months of 35%, 46%, 51%, and 92%, respectively. At 60 months, the highest detection rate (91%) was observed using a combined approach of DUS, CTA, and MRI. However, most of the centres adopt the use of CTA with delayed images as the “gold standard” as it is the most cost-effective single modality for endoleak detection.

Operator experience and appropriate sizing decreased the incidence of type I (both a and b) endoleaks with the use of conventional devices over the years. However, the incidence of type Ia is still high when chimneys are used, reaching sometimes even 30% [14]. Type II endoleaks remain stable over the years in terms of incidence and have been reported around 10% for emergency EVARs and around 20% after elective repairs [15]. Types III–V endoleaks have been reported with lower incidence, in the region of 1–3% [16].

## Symptoms and management

Understanding the aforementioned different pathophysiology mechanisms that lead to the various types of endoleaks permits to distinguish the high- and low-flow nature, which impacts on the requirement or not of repeated intervention and correction. In particular, high-flow endoleaks could lead to the rupture of the aneurysmal sac and may be associated with symptoms like lumbar pain, or even hypotension and tachycardia due to hypovolemic shock [17]. On the other hand, type II endoleaks due to their pathophysiology mechanism of onset are classified as low flow. This is because the sac reperfusion occurs via collateral circulation arising with inverted flow from the periphery towards the aneurysm sac. In these cases, the sac is exposed to a low-pressure flow that may lead to sac enlargement over time. The evolution can be slow and the endoleak can either be managed conservatively [18, 19] or lead to sac expansion over 5 mm in a year in which case treatment will be deemed necessary [20–23]. According to the type of endoleak, different approaches may be followed [24].

## Type I endoleaks

Type Ia may have an immediate onset after graft deployment that offers the option to treat it in the same session. Type Ib usually has a late onset that would require late re-intervention only in case of sac growth.

### Intra-procedure detection

**Fig. 1:**
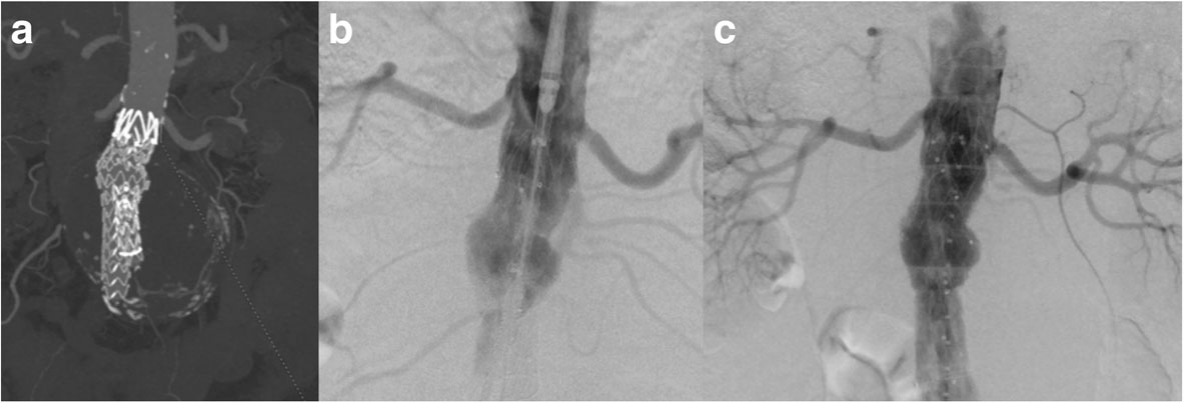
**a** Coronary reconstruction of CTA scan confirming deployment of the graft in a caudal position led to sac expansion in the follow-up period. **b** Angiogram confirmed the low graft position. **c** A cuff was deployed in an immediate infrarenal position to prevent any further sac expansion

**Fig. 2:**
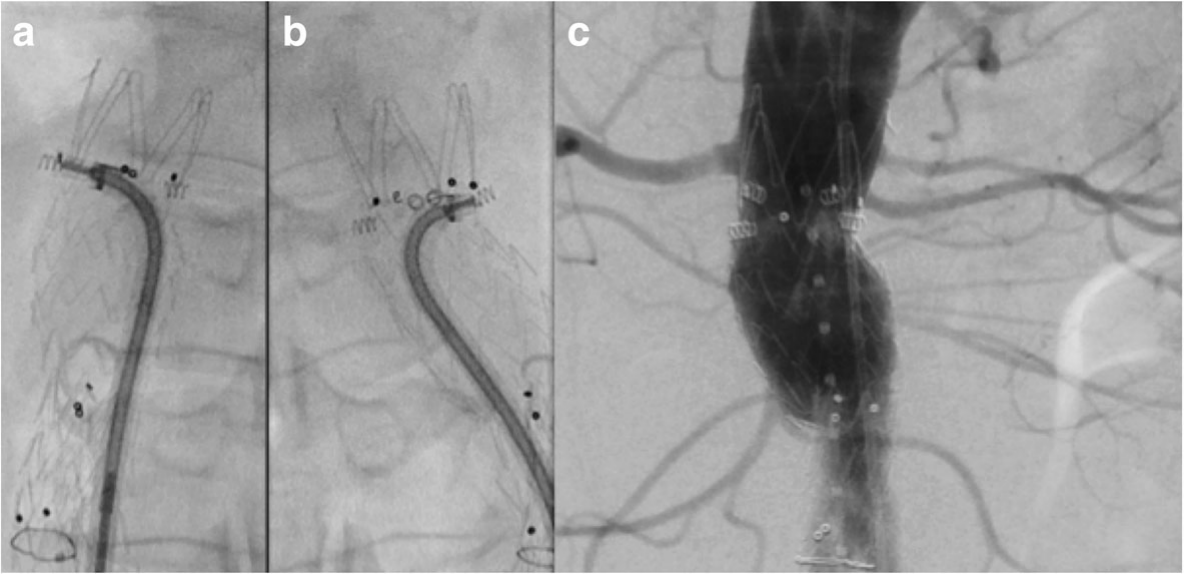
Endoanchors were employed to fix the proximal graft given the short (< 10 mm) neck. **a**, **b** Fluoroscopic picture showing the delivery of the anchors. **c** Angiogram confirming the good apposition of the graft after the deployment of four endoanchors

If a type Ia is detected immediately after graft deployment, the first approach is to expand further the neck of the graft with a moulding balloon. If the effect of the balloon is not satisfactory or if the type Ia is a result of a low graft deployment then the deployment of a cuff needs to be considered (Fig. 1). Cuffs are available for most of the devices; however, if a cuff is not available and the moulding balloon does not appear to work, then the use of a bare stent (Palmaz stent, Cordis Inc) that may expand to the desired diameter needs to be considered. The long-term results of the use of Palmaz stents in treating intra-procedural type Ia endoleak, in patients whom proximal stent graft cuff implantation was not feasible, has been published by Abdulrasak et al. [25] They reported that between 1998 and 2012, 125 patients were treated endovascularly in both elective and emergency settings (83 elective and 42 emergencies) demonstrating a primary and assisted freedom from type Ia endoleak at 5 years of 84 ± 4% and 89 ± 3%, respectively (elective vs emergency cases). Recently, endoanchors have been introduced that may be used to fix further the graft to the aortic wall particularly when the type Ia is due to a short neck [26] (Fig. 2).

### Late detection

If the type Ia is detected in one of the follow-up scans, then all the abovementioned approaches may be equally used according to the anatomy and the operator’s preferences. However, if there is disease progression with neck expansion and no suitable anatomy for a cuff, a Palmaz stent or the use of endoanchors then extension of the graft in the suprarenal and visceral segment is required with the use of more complex chimney/periscope techniques or the use of a fenestrated or branched cuff. The use of chimneys and periscopes are rescuing endovascular techniques that employ the shelf stent grafts to extend the proximal or distal landing zones. Stent grafts are deployed parallel to an aortic extension in order to preserve flow within the aortic visceral vessel branches. Chimneys are performed to divert flow from the aortic lumen towards the branches in a standard anatomy (proximally-distally), whereas periscopes divert flow from the aortic lumen towards the branches in a reversed direction. Montelione et al. [27] reported 12 years experience in 24 patients presenting a type Ia endoleak after a previous endovascular aneurysm repair (EVAR) treated with chimney and/or periscope grafts. They demonstrated a technical success of 96%, with effective intraoperative revascularisation of all target vessels; moreover an estimated survival at 12, 24, and 36 months of 83% and estimated snorkel/chimney patency at the same intervals of 94%. The authors demonstrated aneurysm sac shrinkage during a mean follow-up of 23.4 ± 29 months. Mean maximal aneurysm sac diameter decreased from 88 ± 26 to 85 ± 33 mm (*p* = 0.49) over the course of the follow-up. Aneurysm sac diameter remained stable or decreased in 21 (87%) patients; the other 3 had sac diameter increases > 5 mm, one of which was related to a recurrent type Ia endoleak.

In the case of type Ib endoleaks, embolisation of the ipsilateral internal iliac artery and distal graft extension is usually the most straightforward solution [28, 29].

## Type II endoleaks

### Prevention

Several studies have examined the effectiveness of preoperative embolisation of branch vessels for the prevention of type II endoleaks [30–32]. Alerci et al. [31] evaluated the embolisation of large lumbar arteries prior to EVAR on 124 patients. The rate of type II endoleaks was significantly lower in the embolisation group (3.6% vs 47.8%, *p* < 0.001) after a mean clinical follow-up of 60.5 ± 34.1 months (range 1–144). Piazza et al. [32] reported similar results and also demonstrated that patients who underwent preoperative embolisation experience faster sac shrinkage and that the only independent predictor of a type II endoleak occurrence is preoperative aneurysm sac volume > 125 cm^3^. A meta-analysis by Biancari et al. [33] demonstrated a pooled rate of type II endoleak after IMA embolisation of 19.9% versus 41.4% in patients without IMA embolisation. However, despite this different prevalence of endoleaks, the authors conclude that since treatment for type II endoleaks is needed in less than 20% of cases and this complication can be treated successfully in 60–70% of cases resulting in an aneurysm rupture risk of 0.9%, embolisation of patent IMA may be not of overall benefit to patients undergoing EVAR. Nevertheless, there are groups that still suggest that IMA embolisation may be a preventive measure particularly for arteries with a diameter > 3 mm [34].

Furthermore, the role of intra-procedural embolisation of the aneurysmal sac with thrombin and gelfoam slurry has been investigated, in limited series, demonstrating a trend towards lower rate of type II endoleaks when compared with the literature median rate [35]. Zanchetta et al. [36], in a prospective, nonrandomised pilot study used fibrin glue aneurysm sac embolisation at the time of EVAR in 84 patients and demonstrated a low rate of delayed type II endoleak and a statistically significant decrease in the maximum transverse aneurysm diameter at follow-up. Muthu et al. [37] in a study performed in 2007 where patients that received IMA embolisation combined with intraprocedural thrombin injection in 69 consecutive patients that underwent elective EVAR showed a trend of endoleak reduction; however, no statistically significant difference was reached (26% compared with 14%) and the authors concluded that ongoing research into means to prevent lumbar endoleaks is required. Even though there is a variety of studies available, there is still not enough evidence to support routine embolisation of the IMA or the lumbar arteries or intraprocedural thrombin injection prior to routine EVAR and this remains an operator’s preference [38].

**Fig. 3:**
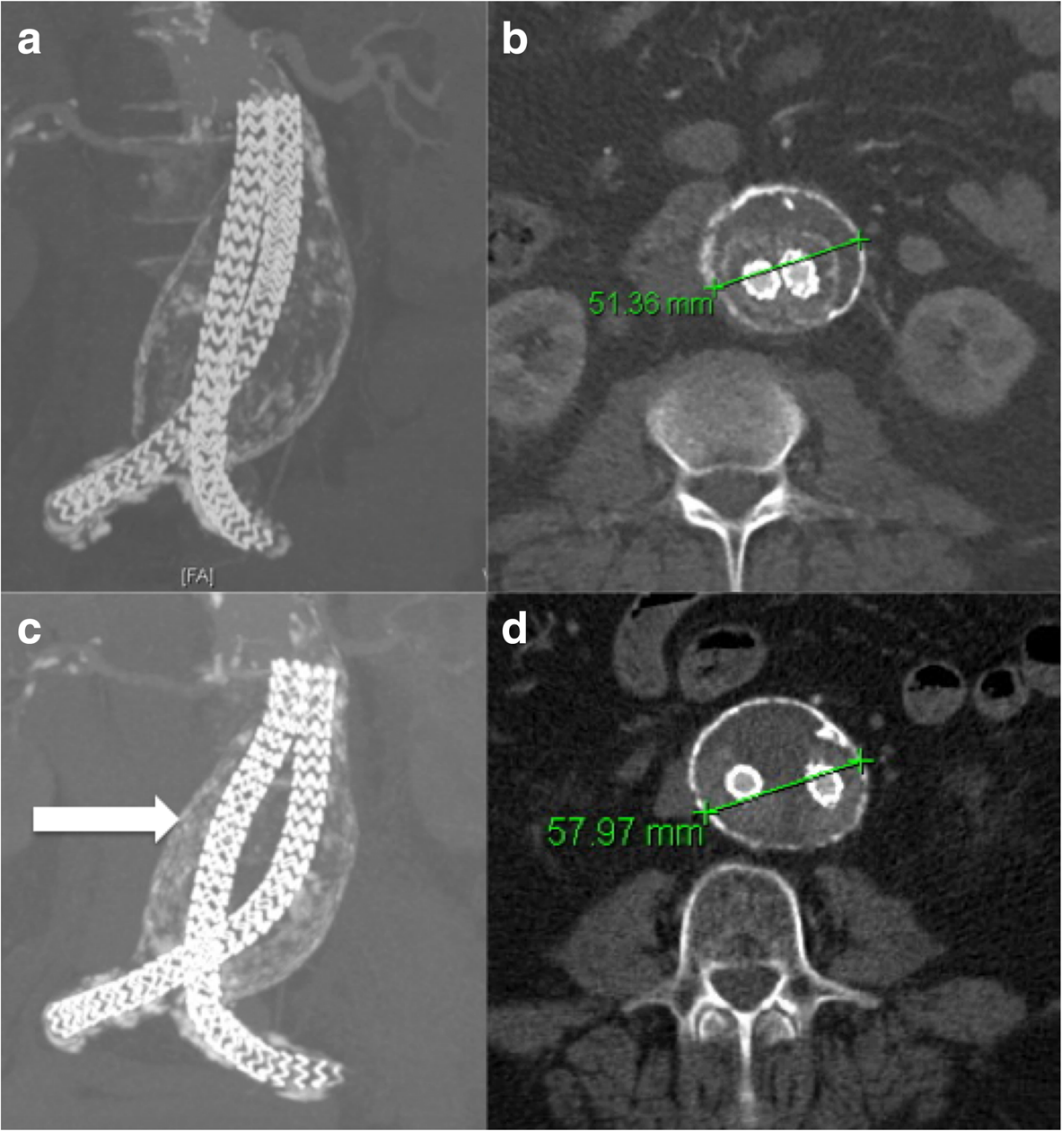
**a** Coronary reconstruction of a CT scan showing the satisfactory deployment of a Nellix device with lack of separation of the grafts. **b** Transverse CT scan confirming the sac size after deployment. **c**, **d** Follow-up CT 2 years later shows separation of the grafts and sac expansion. This is a result of a subtle type Ia endoleak between the two grafts.

To avoid the doubt of preoperative embolisation for the prevention of type II endoleaks, the Nellix EVAS device (Endologics) was developed aiming to “seal” the sac instead of blocking the centripetal flow. The Nellix system comprises two 10-mm balloon expandable chromium-cobalt stent grafts that are inserted into the aorta in a “double-barrel” conformation. A bag is attached to each of the stent grafts and is filled with a polymer during insertion in order to conform according to the anatomical shape of the flow component of the aneurysmal sac [39]. By filling the aortic lumen, the endobags eliminate the space in the sac and limit the possibility of flow towards it. Therefore, when Nellix was launched few years ago, there was a high expectation on limiting the rate of type II endoleaks. Nevertheless, the device did not perform at the standards that it was initially expected, probably because in most of the cases it was used outside the instructions for use (IFU) [40–43]. Specifically, the IFU instructed aortic proximal neck diameter range of 18 to 28 mm, minimum aortic proximal neck length ≥ 10 mm, proximal aortic neck angulation of ≤ 60°, aortic aneurysm with a blood lumen diameter ≤ 70 mm, ratio of maximum aortic aneurysm diameter to maximum aortic blood lumen diameter < 1.4, and distal iliac artery seal zone with length of ≥10 mm and diameter range of 9 to 25 mm. When the device was used within IFU, like in the series of Carpenter et al. [44], it performed much better. However, given the erroneous use, type Ia endoleaks were developed that were impossible to control, leading to graft separation and sac expansion (Fig. 3). Most of the devices were explanted and the graft lost the European Conformity mark in January 2019.

### Endovascular treatment

**Fig. 4:**
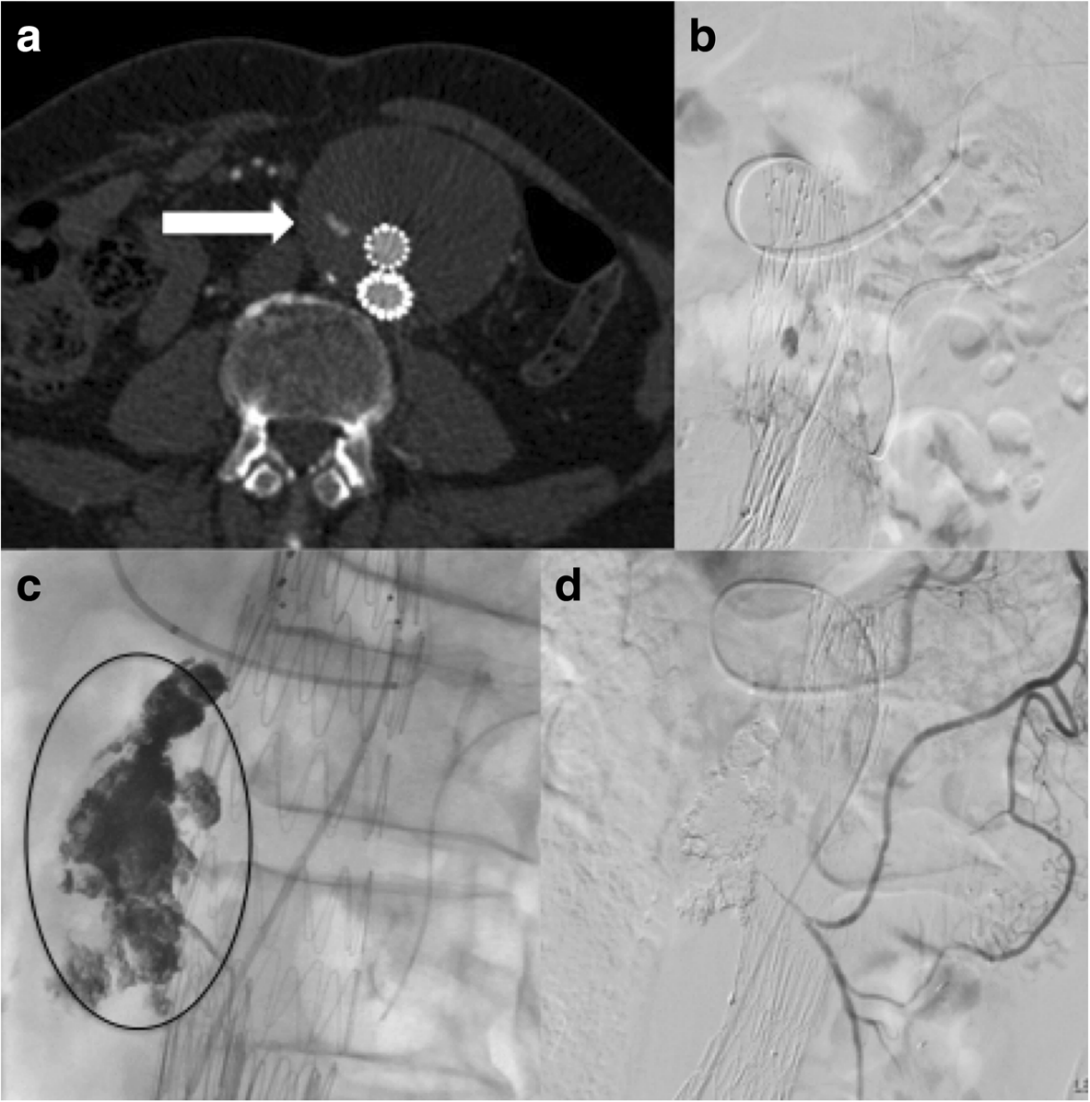
Percutaneous embolisation for type II endoleak. **a** Delayed CTA reveals the presence a type II endoleak (arrow). **b** .Angiogram confirming the access via the Riolan arcade to the IMA. **c** Embolisation with Onyx and (**d**) satisfactory angiographic result

Regarding the endovascular treatment of type II endoleaks, the aim is to reach the aneurysmal sac and block the feeding vessels with embolisation material in order to control the sac growth [45]. The approach differs according to the collateral pathways involved in the sac reperfusion. The focus of treatment is to seal all access to the sac and if possible to directly embolise the sac to avoid recurrence. If the endoleak is supplied by the IMA, a retrograde approach from the superior mesenteric artery and the Riolan arcade will be required (Fig. 4) [46]. In the case of supply via the lumbar arteries, catheterisation via the ileo-lumbar anastomoses that take origin from the ipsilateral internal iliac artery should be performed. The enlargement of this collateral pathway may allow the navigation with a microcatheter permitting super selective catheterisation of the feeding lumbar arteries. Ribè et al. [47] reported a technical success of 100% and no endoleak recurrence via the treated collateral pathways till 19 months of follow-up employing an Onyx liquid embolic agent.

### Percutaneous treatment

**Fig. 5:**
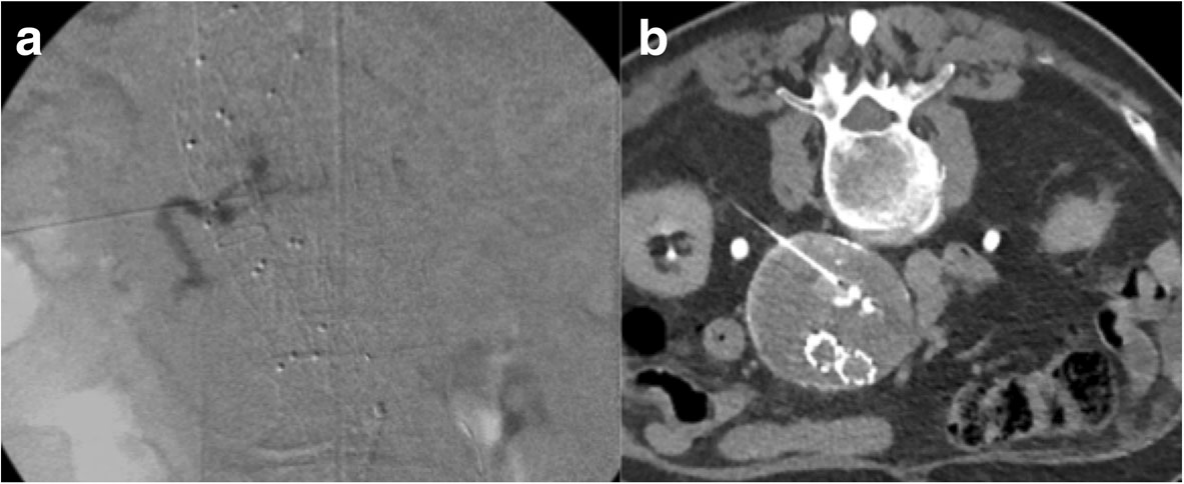
**a** Direct puncture of the sac under CT and fluoroscopic guidance. Angiogram confirms the presence of the small nidus and the feeding vessel. **b** CT scan during embolisation with Onyx

Another way of treating the type II endoleaks is via direct percutaneous sac access under ultrasound (US), computed tomography (CT), or digital subtraction angiography (DSA) guidance (Fig. 5).

Baum et al. [48] described the first experience with translumbar embolisation in literature of type II endoleaks after endovascular repair of abdominal aortic aneurysms. Translumbar paraspinal left-side access with a patient in the prone position is the most common generally performed approach to avoid the inferior vena cava (IVC) while the choice of transabdominal is preferred when an endoleak sac is located or extended anteriorly from the aneurysm sac. Stavropoulos et al. [49] described a prone right-sided percutaneous transcaval approach in order to manage either position of the endoleak or interposed bowel and organs.

The direct access should be performed with a micro puncture access set and guided by ultrasound, CT, or DSA depending on either the anatomy or the operator’s preference. Van Bindsbergen et al. [50] described a technique for real-time three-dimensional needle guidance using cone-beam CT co-registered to fluoroscopy, which can potentially facilitate treatment of small a nidus decreasing radiation and contrast doses compared with traditional methods. Once the nidus is accessed, the needle is exchanged over a guidewire for a vascular sheath and a 4Fr or 5Fr selective catheter. Contrast media is injected to obtain an endoleak-o-gram to define the size of the nidus and the location of the inflow and outflow arteries. Embolisation is performed until the nidus is occluded. The feeding vessels should also be embolised if feasible, but this may be problematic particularly in the case of lumbar arteries due to their unfavourable anatomy as U-turn shape to extend posteriorly [51].

Furthermore, an alternative access route to reach the aneurysmal sac has been reported via the inferior vena cava as mentioned [52–54]. In these cases, the aneurysmal sac is accessed through the inferior vena cava thanks to the employment of a transhepatic shunt access needle set in order to puncture the sac. Then, the microcatheter is advanced within the sac and the embolisation performed. Scali et al. [52] reported experience on transcaval embolisation with coil placement and selective thrombin injection in six patients with 100% technical success and no postoperative complications. In one patient, repeated treatment was required at follow-up of 8.1 months. Giles et al. [54] reported a larger experience in 26 patents who underwent transcaval coil embolisation of the aneurysm sac at a mean of 4.2 ± 4 years after initial endovascular aneurysm repair. There were no procedural adverse events and re-intervention was required in five cases.

A new very interesting percutaneous approach has been reported by Ogawa et al. [55] who performed a transpedicular direct puncture using an isocentre puncture method: an isocentre marker was placed at a site corresponding to the aneurysm sac on fluoroscopy in two directions (frontal and lateral views); then, a vertebroplasty needle was inserted tangentially to the marker under fluoroscopy and advanced to the anterior wall of the vertebral body. Finally a 20 cm-length, 20G needle was inserted through the outer needle of the 13G needle and advanced to the marker.

The embolic agent options include mechanical devices and liquids, used in combination or alone, with a choice of an embolic material tailored to the patient’s anatomy and operator preference. There are no conclusive data on the advantages or disadvantages of the different embolic agents to date, even if high rates of endoleak recurrence (50 %) have been reported in small case series with the use of thrombin as the predominant embolic agent [54], and it seems its use should be avoided.

Some complications of the direct approach have been reported to date, the most clinically significant is a pulmonary embolus secondary to extravasation of the glue in the IVC, stent puncture leading to the development of a type III endoleak [54] and bowel ischemia [52].

### Comparison of endovascular and percutaneous management

A debate in the literature exists between the use of DSPE and transarterial embolisation (TAE) but there is no consensus on which is the most effective or first-line approach for type II endoleaks. Even though there are several retrospective series of patients comparing the two techniques, there is no prospective randomised comparison of TAE versus DSPE. Baum et al. [48] published the first study in 2002 where 20 patients underwent TAE of the IMA and 13 patients underwent direct translumbar embolisation. Over the follow-up period of 254 days, 16 out of the 20 transarterial embolisation, patients required re-intervention due to recanalisation of the initially embolised vessels. Most of the DSPE procedures however were successful with only one recurrence. The authors report a 92% success rate after DSPE using coils, compared with an 80% failure rate of transarterial coil embolisation of the feeding vessels. They concluded that DSPE should be the technique of choice for type II endoleaks. This was a milestone study that triggered the development of further percutaneous techniques.

Sidloff et al. [18] in a systematic review reported an overall failure rate of 37.5 % for TAE compared with a 19% for DSPE; however, it is important to highlight that in many studies, the translumbar approach represents a “bail-out” of the transarterial one and the results would probably have been different if this was the initial approach. On that note, Uthoff et al. [56] conducted a single-centre retrospective analysis of 19 type II endoleaks treated via translumbar approach, and they demonstrated an initial technical success rate of 88%; however, in half of these patients recurrence occurred after 39 months of follow-up and in two-thirds of them a secondary procedure was necessary. In a paper of 2016, Yang et al. [57] reported on twenty-three type II endoleak patients similar sac occlusion effectiveness, between direct sac puncture and transarterial embolisation. The median follow-up was 21.8 months. Direct access was considered as the preferred approach due to shorter fluoroscopic and procedural times. The studies from the literature comparing DSPE and TAE are shown in Table 1.

**Table 1:** Studies in the literature that compare the direct sac with the endovascular approach for the treatment of Type II endoleaks. Technical success*: immediate exclusion of the sac at the first control. Clinical success**: freedom from endoleak recurrence at the follow-up. *DSPE*, direct sac puncture embolisation; *TAE*, transarterial embolisation

Study	No.	Mean follow-up time	Technical success* (%)	Clinical success** (%)
Baum et al. [48]	20 TAE	254 days	90	20
	13 DSPE	254 days	100	92
Stavropoulos et al. [49]	23 TAE	17.3 months	95.7	78.3
	62 DSPE	20.2 months	100	72.6
Nevala et al. [74]	10 TAE	4.5 ± 2.3 years	40	20
	4 DSPE	4.5 ± 2.3 years	100	75
Massis et al. [75]	65 TAE	15 weeks	58	76
	36 DSPE	15 weeks	100	59
Yang et al. [57]	23 DSPE	21.8 months	100	64
			81	57

## Types III, IV, and V endoleaks

### Type III

Chaikof et al. [58] classified first type III endoleaks in 2002 in “Reporting standards for endovascular aortic aneurysm repair”. They may occur either due to disconnection of components of the modular endograft system (IIIa) or a defect in the stent-covering graft fabric (IIIb). Type III b endoleaks are further stratified with respect to the extent of fabric disruption as major (> 2 mm) or minor (< 2 mm). Incidence of type III endoleaks has been reported from 1 to 11% [59]. Also based on the complexity of the anatomy, type IIIa endoleak could be classified as simple and complex. Improper seal, inadequate overlap of modular components, and distal component migration are considered as simple type III endoleaks, whereas major component dislocation including total mal-alignment results in complex type IIIa endoleaks. With the advent of more complex fenestrated EVAR and TEVAR procedures, the total number of junctions in endografts has increased with the possibility of a corresponding increase in the incidence of type III endoleaks. Type III endoleaks arising from side branches are a special concern after fenestrated endografting, with reported rates of 0 to 6.8% [60].

Treatment consists in either realigning the dislodged stent graft parts or advancing another covered stent in the dislodged branches/ fenestrations in the b-EVAR/ f-EVAR case.

### Type IV

In patients who underwent EVAR repair, type IV endoleaks are very rare [61, 62]. Espinosa et al. reported a prevalence of this type of endoleak as 0.3% [63]. Forbes et al. reported the conservative treatment as sufficient, in the case of type IV endoleak. Furthermore, in longer follow-up observation, a type IV endoleak was not the cause of the aneurysm re-supply in open surgery [64] and some clinicians think that type IV endoleaks should be classified as a type V endoleak.

Type IV endoleaks are even more rare with the new-generation stent grafts. In such cases, usually, the best management is surgical conversion with graft explantation [65, 66].

### Type V endoleaks

**Fig. 6:**
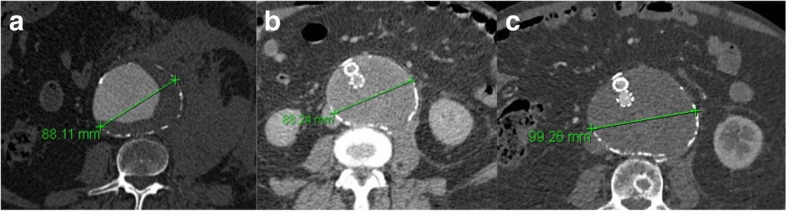
**a-c** CTA scan showing continuous expansion of the aneurysmal sac after initial repair for rupture. The expansion occurred over three years reaching a size of nearly 10 cm but without any evidence of an endoleak. It was considered as a result of “endotension”

Type V endoleaks are a result of “endotension”. With this term is defined the aneurysm sac growth without any detectable endoleak and is a result of increased pressure within the aneurysm sac (Fig. 6) [67]. This may be due to such slow blood flow that it is below the sensitivity limits for detection on current imaging methods. This kind of endoleak seems to be more common with expanded poly-tetrafluoroethylene (ePTFE) fabric grafts rather than polyester covered ones. New-generation ePTFE grafts include a second layer of low permeability ePTFE to decrease this risk [68]. No studies have shown an increased risk of aneurysm rupture among patients with endotension [69, 70]. However, the current view regarding type V management is that when sac growth of more than > 8 mm occurs, then some form of re-intervention is required [71, 72]. Nevertheless, further studies are required to demystify this phenomenon and condition and delineate the appropriate management.

## Future perspectives

Considering the impact that endoleak prevention and treatment have for health economics, there is continuous research on the field with a prediction for an exponential increase in the next 5 years. The main areas that will be developed are the following:

- **Imaging modalities**, mainly for the early detection of and characterisation endoleaks, aiming for radiation-free modalities like contrast-enhanced ultrasound (CEUS) and magnetic resonance imaging. The version 1.3 of the study on the early detection of endoleaks with CEUS (NCT02688751) is recently completed. The primary outcome is to assess the ability of CEUS to detect type I/III endoleaks on CEUS as defined by presence/absence on time-resolved CTA. The secondary outcome is the detection of type II endoleaks and the ability of CEUS to predict the likelihood of a secondary intervention. The study has also assessed healthcare costs related to each imaging modality, considering that EVAR follow-up carries an important economic impact. The results have not been made public yet.

Radiation reduction can also be achieved with dual-energy CT that acquires two different photon spectra in a single acquisition. It can be used to detect endoleaks with good accuracy and at a reduced radiation exposure and some preliminary data is already available [73].

- **Biomarkers** that would predict the aneurysm evolution. The best example is the matrix metalloproteinase (MMP) activity that has been associated with the process of aneurysm development. In essence, if there is a lack of balance between the MMP and its inhibitors, degeneration of the aortic wall is induced. It was previously proved that the serum level of MMP-9 is significantly higher in patients with abdominal aortic aneurysm and in patients with inadequate aneurysm exclusion after EVAR. A multicentre trial of serum levels of MMP-9 as a biomarker of endoleak (NCT01965717) has recently been completed. The aim of the study was to establish the correlation of MMP-9 with specific types of endoleaks and the requirement for re-intervention. The results have not been made public yet.
- **Endostaples** have offered satisfactory results after the completion of the pivotal study of the Aptus Endovascular AAA Repair System (NCT00507559). The ANCHOR (Aneurysm Treatment Using the Heli-FX Aortic Securement System Global Registry) study is currently recruiting patients (NCT01534819) aiming for a primary completion date in 2020. The primary outcome measures are the prevention of graft migration and the treatment of Type Ia endoleak.
- **Navigation systems** in CT offer more accurate needle placement and as the number of direct sac interventions will increase accurate needle placement under CT fluoroscopy will be necessary. The Endoleak Repair Guided by Navigation Technology study (NCT01843322) is a small study of 27 patients that is recently completed and is aiming to delineate whether the treatment of type II endoleaks can be improved by adding navigation technology in terms of precision and reduction of radiation exposure.
- **Novel polymers** will be developed after the Nellix system, regardless of the fact that the results until today have not been as expected. The novel ANEUFIX system in the treatment of endoleaks is assessed in a feasibility study (NCT02487290). The study is a non-randomised, multi-centre safety and feasibility trial of Aneufix ACP-T5 to treat patients with isolated type II endoleaks in the presence of a non-shrinking AAA sac following an EVAR procedure; however, it has only recruited 4 patients at the moment.

## Conclusion

We may conclude that as the treatment options for endovascular repair of abdominal aortic aneurysms and the complexity of devices increase, there will be an increased necessity of prevention and management of endoleaks. Radiology is crucial in the management of such a phenomenon and needs to offer a number of solutions in the endoleak prevention and management.

## Abbreviations

CT: Computed Tomography
DSA: Digital subtraction angiography
DSPE: Direct sac puncture embolisation
DUS: Doppler ultrasound
ePTFE: Expanded poly-tetrafluoroethylene
EVAR: Endovascular aortic repair
EVAS: Endovascular aneurysm sealing
FEVAR: Fenestrated endovascular aortic repair
IMA: Inferior mesenteric artery
IVC: Inferior vena cava
MRI: Magnetic resonance imaging
TAE: Transarterial embolisation
